# Pediatric Gastrointestinal Disorders in Multisystem Inflammatory Syndrome in Children (MIS-C) Associated With COVID-19: A Case Series

**DOI:** 10.1155/crpe/8815325

**Published:** 2025-09-03

**Authors:** Michele di Toma, Ilaria Cassitti, Benedetta Ciccone, Enrica Manca, Alessandra Marinari, Isabella Patisso, Maria Nobili, Angelo Campanozzi

**Affiliations:** ^1^Pediatrics, University Hospital of Foggia, Foggia, Italy; ^2^University of Foggia, Foggia, Italy; ^3^IDESP, UA11-INSERM, University of Montpellier, Montpellier, France; ^4^Pediatric Surgery, University Hospital of Foggia, Foggia, Italy

## Abstract

Multisystem inflammatory syndrome in children (MIS-C) is an immune activation syndrome associated with prior SARS-CoV-2 infection. Clinical manifestations of MIS-C develop 2–6 weeks after SARS-CoV-2 infection with possible involvement of the heart, lungs, kidneys, skin, central nervous system, and digestive tract. Five children with MIS-C (6.6 ± 1.3 years, M:F = 3:2) were admitted to our hospital from January 2021 to March 2022. They all presented with gastrointestinal manifestations, with SARS-CoV-2 molecular swab negativity and positive serology. One child was reported to have a known previous asymptomatic SARS-CoV-2 infection (more than 4 weeks prior to admission). Another one was reported to have received COVID-19 vaccine (second dose four weeks prior to admission). Three/5 were obese children (BMI greater than 95th percentile). All patients experienced fever, abdominal pain, and lack of appetite. Four/5 had vomiting, 3/5 presented diarrhea, 2/5 had constipation, and two male patients had scrotal edema. Three/5 presented with severe gastrointestinal involvement, mimicking appendicopathy; one of them underwent exploratory laparoscopy without histological features of appendicitis. None of them had increased levels of transaminases but one child showed pancreatitis. The median peak value of: IL-6 was 186.5 pg/mL (range: 15.1–692.5; normal values: 0.5–6.4); CRP was 191.4 mg/L (range: 131–386.7; normal values: 0–2); procalcitonin was 19.8 ng/mL (range: 4.27–100; normal value: < 0.5). We treated all patients with intravenous immunoglobulins and steroids. One patient needed oxygen therapy and parenteral nutrition. Nobody died. According to published data, patients with MIS-C have a high rate of abdominal symptoms. Fever and gastrointestinal symptoms were reported in all cases, some of them mimicking acute appendicitis. In the literature, appendectomy was performed in the majority of patients admitted as suspected appendicitis. Moreover, histopathology demonstrated only serosal inflammation, without the typical involvement of acute appendicitis. Following the diagnosis of MIS-C, specific therapy was started, leading to clinical improvement. In conclusion, during the COVID-19 pandemic, MIS-C should always be taken into account in children with persistent fever and severe gastrointestinal symptoms to avoid unnecessary surgical exploration.

## 1. Introduction

SARS-CoV-2 infection was responsible for the pandemic declared in 2020. Most cases of infection were associated with severe symptoms in adults, with higher rates of hospitalization and mortality [1, 2]. In children, a new entity has been observed, characterized by fever, hypotension or shock, abdominal pain, and possible cardiac dysfunction associated with elevated organ damage markers [3, 4] called multisystem inflammatory syndrome in children (MIS-C), also known as pediatric multisystem inflammatory syndrome temporally associated with SARS-CoV-2 (PIMS-TS). This clinical condition manifests itself approximately 2–6 weeks after recovery from SARS-CoV-2 infection [1]. Its symptoms are reported to occur with the following frequency: gastrointestinal (90%), cardiac (80%), mucocutaneous (74%), and respiratory (70%) [1]. Gastrointestinal manifestations at disease onset typically include diarrhea, vomiting, and abdominal pain. MIS-C is associated with an incidence of approximately 3 cases per 10,000 infections, most frequently in the 8–11 age group [2]. This condition is defined according to the guidelines of the World Health Organization (WHO), the Centers for Disease Control and Prevention (CDC), and the Royal College of Paediatrics and Child Health (RCPCH) (Table 1). At the laboratory level, signs of systemic inflammation are observed, with elevated inflammatory markers (erythrocyte sedimentation rate [ESR], C-reactive protein [CRP], D-dimer, ferritin, procalcitonin [PCT], and LDH). These may also be associated with blood cell abnormalities, including lymphopenia, neutropenia, thrombocytopenia, and hypoalbuminemia; thrombosis is sometimes associated. If there is cardiac damage, there is an increase in troponin and NT-proBNP [1]. The treatment regimen involves the administration of immunoglobulins and cortisone [6]. However, in severe cases, the administration of interleukin-1 (IL-1), TNF-alpha, and IL-6 antagonists is necessary [1, 7].

## 2. Case Reports

Between January 2021 and March 2022, five children with MIS-C were admitted to our hospital [8]. Demographic data and clinical characteristics are summarized in Table 2. The mean age of the study population was 6.6 ± 1.3 years. No patients were younger than 4 years or older than 10 years. Males were prevalent, accounting for 60% of the sample. All patients were Caucasian. Sixty percent of patients had a BMI above the 95th percentile. With regard to comorbidities, 80% of the sample had no associated clinical conditions, while one patient (20%) had autism spectrum disorder (ASD). None of the patients had preexisting gastrointestinal diseases. In one case, a previous asymptomatic SARS-CoV-2 infection was documented (more than four weeks prior to admission); only one patient had received the second dose of the COVID-19 vaccine (four weeks prior to admission). All five patients had a previous SARS-CoV-2 infection (serology positive) and a negative nasopharyngeal swab for SARS-CoV-2. The most frequently observed clinical manifestations included fever lasting more than 3 days, conjunctivitis, cardiac involvement (myocardial dysfunction, pericarditis, valvular disease, and increased troponin/proBNP), abdominal pain, and inappetence. Skin rash and mucocutaneous manifestations were present in 80% of cases. Coagulopathy was documented in 40% of patients, while hypotension was present in only one subject. Other gastrointestinal symptoms included diarrhea, constipation, and severe gastrointestinal involvement. One patient also presented with pancreatitis and encephalitis. Scrotal edema was reported in 40% of cases.  Table 3 summarizes the laboratory and imaging characteristics of the patients and their treatment. Analysis of laboratory parameters showed a significant activation of systemic inflammation in all patients. CRP, PCT, and ESR values were significantly elevated. All patients presented with lymphopenia at admission. D-dimer values were elevated in all cases, and consistently, fibrinogen. Myocardial damage markers, troponin and proBNP, were increased in all cases. Furthermore, IL-6 values were significantly increased. All patients presented high titers of anti-SARS-CoV-2 IgG (range: 68.3–109.1 AU/mL, normal values < 8 AU/mL), while only one patient, Case 1, presented high titers of anti-SARS-CoV-2 IgM (32.7 AU/mL, range 0.43–32.7 AU/mL, normal values < 8 AU/mL). Chest X-rays showed four patients with signs suggestive of pulmonary infection or inflammation, such as accentuation of the interstitial pattern, hazy opacities, and atelectatic streaks. Only one patient had a substantially normal radiographic appearance. Echocardiography on admission showed abnormalities in three patients: two had valvular regurgitation (mitral and tricuspid) associated with pericardial effusion, one of whom had circumferential pericardial effusion; one patient had reduced ejection fraction (EF) (< 50%). At follow-up, all patients showed recovery of cardiac function with normalization of echocardiographic findings. Only one patient underwent diagnostic laparoscopy, during which an appendectomy was performed and peritoneal fluid was collected for culture, with negative results. Histological examination revealed nonspecific inflammation of the appendix. All patients received intravenous immunoglobulin (IVIG) and corticosteroids. Antiplatelet therapy was administered in two cases, while anticoagulant therapy with enoxaparin was used in two patients with D-dimer values greater than 5 times the limit value, considering the procoagulant risk associated with MIS-C. Broad-spectrum antibiotic therapy was administered to all patients. Only patient number 5 required support with diuretics, oxygen therapy, and total parenteral nutrition (TPN) due to the higher severity of the clinical condition.

## 3. Discussion

MIS-C often manifests clinically in a manner similar to other childhood diseases, including Kawasaki disease. However, it differs in terms of age of onset, ethnicity, previous comorbidities, and risk factors: MIS-C occurs in school-aged children (8–11 years) and is more prevalent in African and Spanish populations; Kawasaki disease occurs more frequently in preschool children (6 months–5 years) and is more prevalent in Asian populations [1, 5, 7]. Other authors describe a wider age range for the onset of MIS-C, between 6 and 12 years, with a prevalence in males, attributing obesity as the main preexisting comorbidity [9]. In line with these findings, our patients ranged in age from 5 to 8 years. However, MIS-C remains a rare condition, which shares some treatment protocols with Kawasaki disease, such as the use of IVIG and corticosteroids. The predisposing factor for MIS-C remains unclear, although a stronger association with obesity/overweight, respiratory, or cardiac conditions has been confirmed [1]. Our three obese and one overweight patients had higher inflammatory markers and more severe multisystem involvement. It has been suggested that MIS-C is caused by a dysregulation of the autoimmune system, triggered by a delayed cytokine storm due to the ability of SARS-CoV-2 to block type I and type III interferon responses [1, 3, 7]. Another explanation for the pathogenesis of MIS-C involves the role of SARS-CoV-2-specific IgG, which is temporally associated with the onset of symptoms [2]. Children with MIS-C produce high titers of anti-SARS-CoV-2 IgG antibodies; according to published data, this may reflect local inflammation, particularly in the gastrointestinal mucosa, induced by intestinal SARS-CoV-2 infection [10]. MIS-C is associated with a higher prevalence of gastrointestinal symptoms [11–13]. This characteristic may be due to the primary site of infection being the gastrointestinal tract [1, 7]. The classic gastrointestinal symptoms are abdominal pain, vomiting, and diarrhea [14, 15]. All our patients progressively developed multiorgan involvement starting with abdominal pain. Almost half of patients with MIS-C have severe gastrointestinal involvement [16]. The literature agrees that MIS-C with gastrointestinal involvement can present with acute abdomen [17, 18]. In most cases of acute abdomen, surgical exploration was necessary [19]. Given the difficulty in differentiating MIS-C from acute appendicitis, diagnostic scores have been proposed [11, 20, 21]. Other authors suggest that MIS-C should be suspected in all cases of acute appendicitis with marked increase in inflammation markers, persistent fever, and involvement of other organs, encouraging testing for SARS-CoV-2 [22, 23]. Okarska-Napierała et al. [24] showed in a cross-sectional study of 21 children with MIS-C who underwent appendectomy that histology revealed no edema or neutrophil infiltration typical of acute appendicitis; similar results were produced by Hwang et al. [25]. Consistent with these findings, one of our patients underwent exploratory laparoscopy without histological features of appendicitis. Moreover, a clinical picture characterized by appendicitis and pancreatitis has also been described [26]. There are also cases in the literature of severe necrotizing pancreatitis in patients with MIS-C who required IVIG and steroid therapy [27]. Finally, ultrasonography is a helpful exam, detecting intra-abdominal free fluid strongly associated with severe clinical outcomes in MIS-C patients [28]. Although the presentation of MIS-C mimicking acute abdomen is well-described, this case series serves to concretely reinforce these findings and underscores the importance of considering MIS-C in the differential diagnosis.

## 4. Conclusions

Although MIS-C is not a frequent condition, it requires considerable resources for early diagnosis, especially in the absence of previously known SARS-CoV-2 infection. The gastrointestinal symptoms, which are already common in pediatric diseases, represent a challenge both in diagnosis and management of patients with MIS-C. The severity of the clinical presentations had justified in an initial phase, the frequent use of surgical procedures, but only the use of specific MIS-C therapy was able to significantly improve the outcome. In conclusion, children with acute abdomen during the COVID-19 pandemic required an early diagnosis of MIS-C to avoid unnecessary surgery.

## Figures and Tables

**Table 1:** Definitions of COVID-19–related multisystem inflammatory syndrome [5].

Feature	Source of definition			
	RCPCH	CDC	WHO	CPSP
Name of syndrome	Pediatric multisystem inflammatory syndrome temporally associated with COVID-19 (PIMS-TS)	Multisystem inflammatory syndrome in children (MIS-C)	Multisystem inflammatory syndrome in children (MIS-C)	Pediatric inflammatory multisystem syndrome temporally associated with COVID-19 (PIMS-TS)
Fever	Persistent at > 38.5°C	> 38°C or subjective ≥ 24 h	≥ 3 days	> 38°C for ≥ 3 days
Age	Not specified “child”	< 21 years	0–19 years	< 18 years
Multisystem	Single organ or multiorgan dysfunction and additional features	≥ 2 organ systems	≥ 2 features	Not specific but implicit
Laboratory features	Neutrophilia, lymphopenia, and elevated CRP	One or more the following: elevated CRP, ESR, fibrinogen, procalcitonin, D-dimer, ferritin, LDH, IL-6, neutrophilia, lymphopenia, and low albumin	Elevated ESR, CRP, or procalcitonin	Elevated CRP, ESR, or ferritin
Excludes other causes	No	No	Yes	No
SARS-CoV-2 PCR, antibodies, or exposure necessary	No	Yes	Yes	No

**Table 2:** Demographic and clinical characteristics (*N* = 5).

Characteristics	Value, *N* (%)
*Age (years)*	
< 4	0 (0)
5–6	2 (40)
7–8	2 (40)
9–10	1 (20)
> 10	0

*Sex*	
Male	3 (60)
Female	2 (40)

*Ethnicity*	
Caucasian	5 (100)
Others	0 (0)

*BMI*	
< 25th pc	0 (0)
25th–50th pc	1 (20)
50th–90th pc	0
90th–95th pc	1 (20)
> 95th pc	3 (60)

*Comorbidity*	
None	4 (80)
ASD (autism spectrum disorder)	1 (20)

*Clinical characteristics*	
Fever > 3 days	5 (100)
Rash	4 (80)
Conjunctivitis	5 (100)
Mucocutaneous lesions	4 (80)
Hypotension	1 (20)
Cardiac involvement	5 (100)
Coagulopathies	2 (40)
Abdominal pain	5 (100)
Loss of appetite	5 (100)
Diarrhea	3 (60)
Constipation	2 (40)
Severe GI involvement	4 (80)
Scrotal edema	2 (40)
Pancreatitis	1 (20)
Appendiceal disease	3 (60)
Encephalitis	1 (20)

**Table 3:** Laboratory and imaging characteristics.

	Case 1 (M, 5 years, BMI > 95th pc)	Case 2 (M, 5.5 years, BMI 25th–50th pc)	Case 3 (F, 6.9 years, BMI > 95th pc)	Case 4 (M, 8.9 years, BMI > 95th pc)	Case 5 (F, 6.8 years, BMI 90th–95th pc)	Median (range)	Normal values
CRP (mg/L)	161.4	131	386.7	191.4	225.2	131–386.7	< 2
PCT (ng/mL)	9.2	86.4	19.8	2.4	> 100	2.4–> 100	< 0.5
ERS (mm/h)	38	49	40	36	37	36–49	
WBC (/μL) first sample	5710	19,060	9080	8350	7620	5710–19,060	6000–18000
Lymphocytes first sample (/μL)	760	1820	430	460	580	430–1820	1800–13500
D-dimer (ng/mL)	2384	2662	2233	1922	3081	1922–3081	0–500
Fibrinogen (mg/dL)	587	630	1038	656	525	525–1038	200–450
Troponin (ng/L)	60.5	48.3	70	34.3	105.2	34.3–105.2	< 19.8
NT-proBNP (pg/mL)	20,400	9500	20,200	2480	26,000	2480–26,000	< 300
IL-6 (pg/mL)	470.1	15.1	186.5	74.4	692.5	15.1–692.5	< 6.4
Anti-SARS-CoV-2 IgG (AU/mL)	190.1	158.8	98	129	68.3	68.3–190.1	< 8
Anti-SARS-CoV-2 IgM (AU/mL)	32.7	5.1	4.7	4.43	0.43	0.43–32.7	< 8
Chest X-ray	Increased reticular pattern in the perihilar and parahilar regions	Some consolidations in the parahilar region	Left lower parahilar bronchovascular crowding with subtle atelectasis	Consolidation in the right perihilar and parahilar region	Increased reticular pattern in the perihilar and parahilar regions	—	—
Echocardiogram (first evaluation)	EF < 50%	Normal	Mild mitral and tricuspid insufficiency; minimal pericardial effusion	Mild mitral and tricuspid insufficiency; minimal pericardial effusion	EF < 55%, mild mitral insufficiency, moderate tricuspid insufficiency	—	—
Echocardiogram (follow-up)	Normal	Normal	Normal	Normal	Normal	—	—
Exploratory laparoscopy	+	−	−	−	−	−	−
*Treatment*							
IGIV	+	+	+	+	+		
Corticosteroids	+	+	+	+	+		
Aspirin	−	+	−	−	+		
Heparin	+	−	−	−	+		
Antibiotics	+	+	+	+	+		
Diuretics	−	−	−	−	+		
Oxygen	−	−	−	−	+		
TPN	−	−	−	−	+		

## Data Availability

The data that support the findings of this study are available from the corresponding author upon reasonable request.

## References

[1] Patel J. M. (2022). Multisystem Inflammatory Syndrome in Children (MIS-C). *Current Allergy and Asthma Reports*.

[2] Payne A. B., Gilani Z., Godfred-Cato S. (2021). Incidence of Multisystem Inflammatory Syndrome in Children Among US Persons Infected With SARS-CoV-2. *JAMA Network Open*.

[3] Rowley A. H. (2020). Understanding SARS-CoV-2-Related Multisystem Inflammatory Syndrome in Children. *Nature Reviews Immunology*.

[4] Santos M. O., Gonçalves L. C., Silva P. A. N. (2022). Multisystem Inflammatory Syndrome (MIS-C): A Systematic Review and Meta-Analysis of Clinical Characteristics, Treatment, and Outcomes. *The Journal of Pediatrics*.

[5] Tam H., El Tal T., Go E., Yeung R. S. M. (2020). Pediatric Inflammatory Multisystem Syndrome Temporally Associated With COVID-19: A Spectrum of Diseases With Many Names. *Canadian Medical Association Journal*.

[6] Phan P. H., Hoang C. N., Nguyen H. T. T., Cao T. V., Le C. Q., Tran D. M. (2025). Comparison of Methylprednisolone Alone Versus Intravenous Immunoglobulin Plus Methylprednisolone for Multisystem Inflammatory Syndrome in Children (MIS-C). *BMJ Paediatrics Open*.

[7] Rowley A. H. (2021). Diagnosing Severe Acute Respiratory Syndrome Coronavirus 2 (SARS-CoV-2) Related Multisystem Inflammatory Syndrome in Children (MIS-C): Focus on the Gastrointestinal Tract and the Myocardium. *Clinical Infectious Diseases*.

[8] (2022). ESPGHAN 54th Annual Meeting Abstracts. *Journal of Pediatric Gastroenterology and Nutrition*.

[9] Dufort E. M., Koumans E. H., Chow E. J. (2020). Multisystem Inflammatory Syndrome in Children in New York State. *New England Journal of Medicine*.

[10] Thiriard A., Meyer B., Eberhardt C. S. (2023). Antibody Response in Children With Multisystem Inflammatory Syndrome Related to COVID-19 (MIS-C) Compared to Children With Uncomplicated COVID-19. *Frontiers in Immunology*.

[11] Toker Kurtmen B., Ekemen Keles Y., Tekindal M. A., Koyluoglu G., Yilmaz Ciftdogan D. (2023). Differentiating Abdominal Pain due to COVID-19 Associated Multisystem Inflammatory Syndrome From Children With Acute Appendicitis: A Score System. *Pediatric Surgery International*.

[12] Whittaker E., Bamford A., Kenny J. (2020). Clinical Characteristics of 58 Children With a Pediatric Inflammatory Multisystem Syndrome Temporally Associated With SARS-CoV-2. *JAMA*.

[13] Saalabian K., Rolle U., Friedmacher F. (2021). Impact of the Global COVID-19 Pandemic on the Incidence, Presentation, and Management of Pediatric Appendicitis: Lessons Learned From the First Wave. *European Journal of Pediatric Surgery*.

[14] Coles V., Yardley I., Hameed S., Brennan K. (2021). Paediatric Post-COVID-19 Hyperinflammatory Syndrome Mimicking Appendicitis: A Case Series. *Annals of the Royal College of Surgeons of England*.

[15] Boybeyi-Turer O., Ozsurekci Y., Gurlevik S. L., Oygar P. D., Soyer T., Tanyel F. C. (2022). Management of Acute Abdomen During the Active Disease Course of COVID-19 and Multisystem Inflammatory Syndrome in Children. *Surgery Today*.

[16] Unny A. K., Rajashree P., Sundararajan L., Sankar J. (2022). Abdominal Manifestations of Multisystem Inflammatory Syndrome in Children: A Single-Center Experience. *Indian Pediatrics*.

[17] Serra A. M., Ventura A. M. C., Xavier L. F., Simões A. B., Duarte-Neto A. N. (2021). SARS-CoV-2 Identification in an Acute Appendicitis Case: Acute Abdomen as Manifestation of Multisystem Inflammatory Syndrome in a Child With COVID-19. *Brazilian Journal of Infectious Diseases*.

[18] Olmos García J. M., Pareja Marín F., Martínez Bayo Á., Silvestre Beneyto R., Escrivá Tomás P. (2021). Acute Appendicitis in Children With Multisystemic Inflammatory Syndrome Associated to SARS-CoV-2 (MIS-C): A Complication to Consider. *Anales de Pediatría (English Edition)*.

[19] Yock-Corrales A., Lenzi J., Ulloa-Gutiérrez R. (2021). Acute Abdomen and Appendicitis in 1010 Pediatric Patients With COVID-19 or MIS-C: A Multinational Experience From Latin America. *The Pediatric Infectious Disease Journal*.

[20] Hegde B. N., Slater B. J. (2022). Long-Term Effect of the COVID-19 Pandemic on the Management of Pediatric Appendicitis. *Pediatric Annals*.

[21] Falqueto L. E., Vissoci C. M., Ferreira I. C. B. (2021). COVID-19 Associated Multisystem Inflammatory Syndrome in Children Mimicking Acute Appendicitis: How to Differentiate and Conduct Pediatric Patients During the Pandemic?—Proposal of a Management Flowchart. *Revista do Colégio Brasileiro de Cirurgiões*.

[22] Vansevičienė I., Krunkaitytė U., Dekerytė I. (2022). Does Multisystem Inflammatory Syndrome Only Mimic Acute Appendicitis in Children or Can It Coexist: When Should We Suspect MIS-C?. *Medicina (Kaunas)*.

[23] Azılı M. N., Güney D., Oztorun C. I. (2022). Determination of Factors to Distinguish MIS-C From Acute Appendicitis in Children With Acute Abdominal Pain. *European Journal of Pediatric Surgery*.

[24] Okarska-Napierała M., Woźniak W., Mańdziuk J. (2024). Pathologic Analysis of Twenty-One Appendices From Children With Multisystem Inflammatory Syndrome Compared to Specimens of Acute Appendicitis: A Cross-Sectional Study. *The Pediatric Infectious Disease Journal*.

[25] Hwang M., Wilson K., Wendt L. (2021). The Great Gut Mimicker: A Case Report of MIS-C and Appendicitis Clinical Presentation Overlap in a Teenage Patient. *BMC Pediatrics*.

[26] Kareva L., Stavrik K., Mironska K., Hasani A., Bojadzieva S., Shuntov N. C. (2021). A Case of Multisystem Inflammatory Syndrome in Children Presenting as Acute Appendicitis and Pancreatitis. *Pril (Makedon Akad Nauk Umet Odd Med Nauki)*.

[27] Distel H., Hutchins K., Purohit P. J., Bégué R. E. (2024). Multisystem Inflammatory Syndrome in Children Presenting as Acute Severe Necrotizing Pancreatitis: A Case Report. *JPGN Reports*.

[28] Yasar Y., Coskun M., Yasar E. (2025). The Association Between Abdominal Ultrasound Findings and Clinical Severity in MIS-C Children With Extracardiac Symptoms. *European Journal of Pediatrics*.

